# In Silico Discovery of a Substituted 6-Methoxy-quinaldine with Leishmanicidal Activity in *Leishmania infantum*

**DOI:** 10.3390/molecules23040772

**Published:** 2018-03-27

**Authors:** Strahinja Stevanović, Andrej Perdih, Milan Senćanski, Sanja Glišić, Margarida Duarte, Ana M. Tomás, Filipa V. Sena, Filipe M. Sousa, Manuela M. Pereira, Tom Solmajer

**Affiliations:** 1 Center of Multidisciplinary Research, Institute of Nuclear Sciences “Vinča”, University of Belgrade, 11001 Belgrade, Serbia; sencanski@vin.bg.ac.rs (M.S.); sanja@vinca.rs (S.G.); 2 National Institute of Chemistry, Hajdrihova 19, 1001 Ljubljana, Slovenia; tom.solmajer@ki.si; 3 i3S-Instituto de Investigação e Inovação em Saúde, Universidade do Porto and IBMC-Institute for Molecular and Cell Biology, 4099-002 Porto, Portugal; mduarte@ibmc.up.pt (M.D.); atomas@ibmc.up.pt (A.M.T.); 4 ICBAS, Instituto de Ciências Biomédicas Abel Salazar, Universidade do Porto, 4099-002 Porto, Portugal; 5 Instituto de Tecnologia Química e Biológica António Xavier, Universidade Nova de Lisboa, 1099-085 Oeiras, Portugal; fsena@itqb.unl.pt (F.V.S.); filipe.sousa@itqb.unl.pt (F.M.S.); mpereira@itqb.unl.pt (M.M.P.); 6 University of Lisbon, Faculty of Sciences, BioISI-Biosystems & Integrative Sciences Institute, Campo Grande, C8, 1749-016 Lisboa, Portugal

**Keywords:** *Leishmania infantum* alternative NADH dehydrogenase (*Li*NDH2), antileishmanial drugs, *Leishmania infantum* virtual screening, drug design

## Abstract

There is an urgent need for the discovery of new antileishmanial drugs with a new mechanism of action. Type 2 NADH dehydrogenase from *Leishmania infantum* (*Li*NDH2) is an enzyme of the parasite’s respiratory system, which catalyzes the electron transfer from NADH to ubiquinone without coupled proton pumping. In previous studies of the related NADH: ubiquinone oxidoreductase crystal structure from *Saccharomyces cerevisiae*, two ubiquinone-binding sites (UQ_I_ and UQ_II_) were identified and shown to play an important role in the NDH-2-catalyzed oxidoreduction reaction. Based on the available structural data, we developed a three-dimensional structural model of *Li*NDH2 using homology detection methods and performed an in silico virtual screening campaign to search for potential inhibitors targeting the *Li*NDH2 ubiquinone-binding site 1–UQ_I_. Selected compounds displaying favorable properties in the computational screening experiments were assayed for inhibitory activity in the structurally similar recombinant NDH-2 from *S. aureus* and leishmanicidal activity was determined in the wild-type axenic amastigotes and promastigotes of *L. infantum*. The identified compound, a substituted 6-methoxy-quinaldine, showed promising nanomolar leishmanicidal activity on wild-type axenic promastigotes and amastigotes of *L. infantum* and the potential for further development.

## 1. Introduction

The leishmaniases, classified as neglected tropical diseases, comprise a group of diseases caused by more than 20 known species of protozoan *Leishmania* parasites that are usually transmitted to humans through bites of infected female phlebotomine sandflies. There are three main forms of leishmaniasis, described as cutaneous, visceral or kala-azar, and mucocutaneous. The disease predominantly affects low-income countries with an overall population of 200 million people in Asia, Africa, South and Central America and is commonly associated with malnutrition, substandard housing conditions, and lack of resources [1].

The enzyme family of type 2 NADH:quinone oxidoreductases (NDH-2s) is present in various microorganisms and plants, and a possible variation in humans [2,3]. These enzymes are putative drug targets for the treatment of parasitic infections such as leishmaniasis as these parasites also present a NDH-2, *Li*NDH2 in *L. infantum* [4]. In general, NDH-2 is a peripheral membrane protein which forms an intimate dimer, in which packing of the monomeric units within the dimer creates an amphiphilic membrane–anchor domain structure [5].

**Figure 1:**
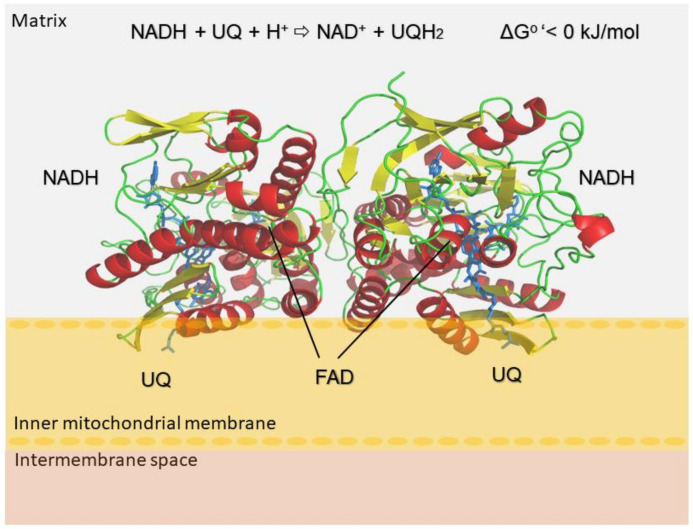
Oxidoreduction reaction of NDH-2 enzymes. Cartoon representation of the model structure of the NDH-2 from *L. infantum*. The homodimer representation of *Li*NDH2 homology model generated using Phyre2 server is presented in a cartoon style. Orientation and structural arrangement of the monomer units were obtained using 3D alignment with the used template structures (Protein Data Bank IDs: 4g6g, 4g73 and 5jwa [9,10]). The homodimer consists of reciprocally oriented monomers and is located on the surface of the mitochondria intermembrane. C-termini are embedded into the membrane layer (inner mitochondrial membrane). In depicted reaction: NADH/NAD^+^ denote nicotinamide adenine dinucleotide protonated/deprotonated; UQ/UQH_2_ ubiquinone deprotonated/protonated and FAD flavin adenine dinucleotide.

The oxidoreduction mechanism of NDH-2 enzymes is explained through the electron transfer process from soluble NADH via flavin adenine dinucleotide (FAD) to membrane-bound UQ, a catalytic reaction shown in the top section of Figure 1. In comparison to the type 1 NADH: quinone oxidoreductase family, the respiratory complex I, which also catalyzes electron transfer reactions and is ubiquitously present in animals and other species, no proton motive force is observed and the process of catalysis is irreversible and exergonic [6,7]. The energy of the NDH-2 catalyzed reaction is released as heat and produced ubiquinol is then reoxidized by other enzymes, such as the alternative oxidase. This enzyme is directly responsible for a reduction of oxygen, suggesting that both enzymes are crucial for the sustained respiration cycle of the parasite [8].

The enzymatic mechanism of NDH-2s was proposed to follow a two-site ping-pong mechanism, but recent evidence showed the presence of a ternary complex [11,12]. Two different non-overlapping pockets for UQ and NADH were reported [6]. Additionally, from the obtained crystal structure of *Sc*NDH-2, two ubiquinone binding sites (UQ_I_ and UQ_II_) were suggested (Figure S2A). A charge complex between NAD^+^ and FAD was observed, which was only dissociated by quinone reduction [13]. Based on available structural data [9], including the observation that the UQ_I_ binding site is also showing a higher percentage of the electron density in comparison with the UQ_II_, and is thus better defined through crystallographic structure analysis (Figure S2B), UQ_I_ appears thus to be more appropriate for structure-based design studies in search for new NDH-2 inhibitors. Inhibition of the *L. infantum* NDH-2 enzyme should cause respiration difficulties and possible parasite elimination [7].

In this study, we have developed a homology model of the *Li*NDH2 and performed a virtual screening to search for potential inhibitors against the *Li*NDH2 targeting UQ_I_. Selected compounds displaying favorable properties as a result of in silico screening were assayed for their in vitro inhibitory activity in the available structurally analogous *Sa*NDH2 and their leishmanicidal potential in *L. infantum* axenic amastigotes and promastigotes.

## 2. Results and Discussion

### 2.1. Construction of the LiNDH2 Homology Model

**Figure 2:**
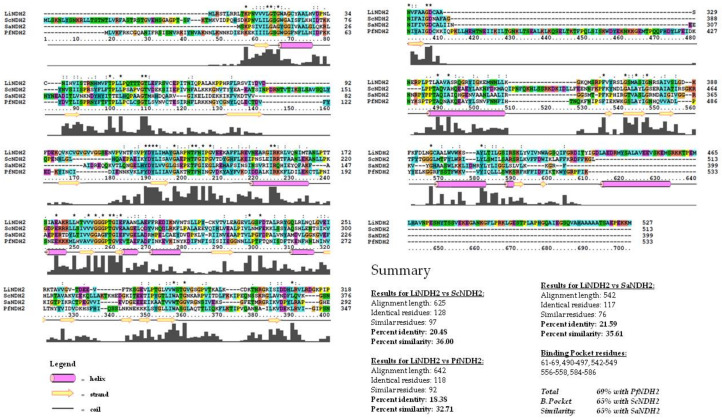
Sequence alignment of query (model–*Li*NDH2) and important template sequences: *Sc*NDH2, *Pf*NDH2 and *Sa*NDH2. Secondary structure prediction within alignment was generated using PSIPRED v3.3 [18]. For further information regarding secondary structure alignment (SSA), see Figure S1 [19]. Summary in the lower right corner contains the alignment scores between model and each template, sequence identity and percent similarity (homology). The alignment analysis in the summary is obtained using Sequence Manipulation Suite [20]. Within summary, numeration of amino acid residues which create the binding pocket is deduced from sequence alignment and the total binding pocket similarity was calculated considering just these residues. *Li*, *Leishmania infantum*; *Sc*, *Saccharomyces cerevisiae*; *Pf*, *Plasmodium falciparum*; *Sa*, *Staphylococcus aureus*.

Due to unavailable crystallographic data on *Li*NDH2, we used a homology modeling approach for the generation of the three-dimensional target structure. For this purpose, we utilized Phyre 2 web-portal v2.0 [14]. The best identified template structures found in the Protein Data Bank (PDB) were rotenone-insensitive NADH:ubiquinone oxidoreductase from *S. cerevisiae* (Chain B, 4g6g or 4g73)—*Sc*NDH2 and NADH:quinone oxidoreductase from *S. aureus* (Chain C, 4xdb)—*Sa*NDH2 [9,15,16]. The modeling pipeline of Phyre2 web-portal produced a structure with 100% modeling confidence and a score of 33% and 27% identity for *Sc*NDH2 and *Sa*NDH2, respectively, (Table S1). The modeling confidence suggests that our model is likely to adopt the overall 3D structure, generated with the Phyre2 pipeline. Also, there is strong evolutionary relatedness between the query (*Li*NDH2) and the template structures (*Sc*NDH2 and *Sa*NDH2). Although sequence identity is above the twilight zone [17], we find that further sequence analysis using primary sequence alignment methods might lead to more useful data, especially regarding amino acids’ similarity within the UQI binding pocket which was used in virtual screening. For this purpose, we compared the results of the Phyre2 alignment method with Clustal alignment, as outlined in Figure 2. The most prominent template for the *Li*NDH2 homology model development was *Sc*NDH2, as shown using primary sequence alignment. The next best template was *Sa*NDH2, and we used both structures in the development of our homology model which was important for the design of in vitro evaluation. During the course of writing this paper, another NDH-2–*Pf*NDH2 was submitted to the PDB (5jwa) [10]. We found these data important for sequence alignment purposes and included them in the analysis. Two conserved regions between residues 50–70 are substrate-binding residues. Motif GxGxxG is found in each homolog and in our model sequence. Another conserved domain is located between residues 175–195. These are twisted beta sheets, which are most likely responsible for the FAD binding. The binding site UQ_I_ differs in some extent between *Li*NDH2 and other NDH-2s. More details are derived from sequence alignment and summarized in Figure 2.

**Figure 3:**
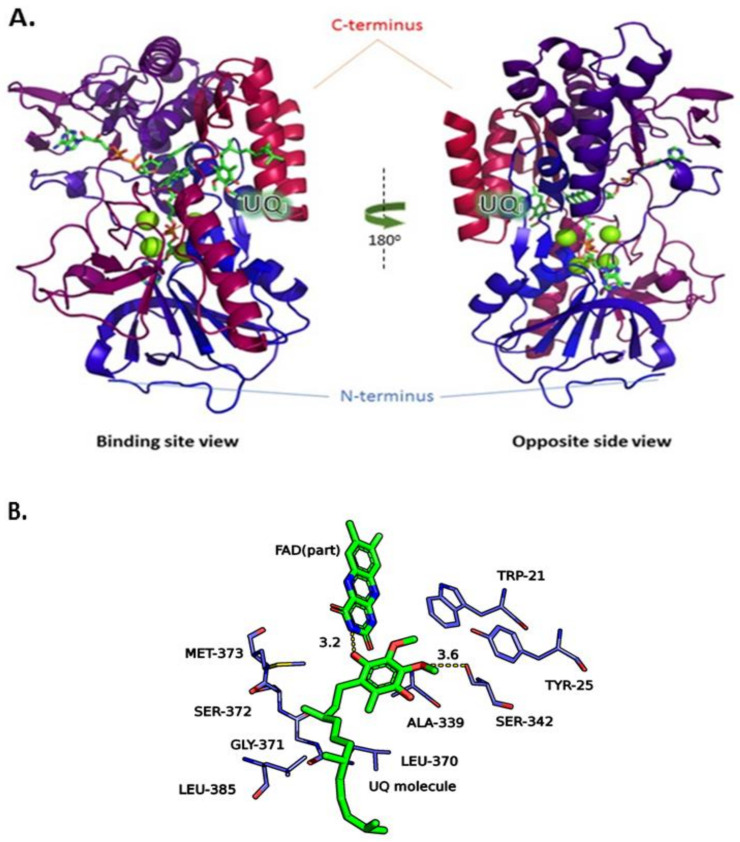
(**A**) Cartoon representation of the homology structural model of *Li*NDH2 in two orientations. Color code: C-terminus to N-terminus (red to blue); Mg^2+^ ions (green spheres); NADH, flavin adenine dinucleotide (FAD) and ubiquinone-binding (UQ) molecules are colored by atom element; (**B**) close up on UQ_I_ (docking data) with flavin group, UQ molecule and residues within 5 Å radius. Interactions with UQ to FAD, 3.3 Å and UQ to Ser342, 3.4 Å are shown.

The main difference between the quinone binding site from *Li*NDH2 and that of *Sa*NDH2 (and other templates as well) is the lack of the AQxAxQ motif. The two glutamine residues present in this motif may have a role in quinone binding, as discussed elsewhere [2,13,15]. Overall, the structural sequence alignment also shows significant percentage of similarity between the active sites of *Li*NDH2 and *Pf*NDH2. Residues Ser342 and Tyr25 are unique to *Li*NDH2 sequence, being part of the UQ_I_ binding site. Moreover, the model of *Li*NDH2 shows higher percentage of hydrophobic residues in comparison to all template structures and we found this to be the main source of divergence among NDH-2s. Such an observation has repercussions in formation of the binding hydrophobic cavity within UQ_I_, namely residues between Arg368–Met373 and Val383–Leu385, thus indicating that selectivity for inhibitor development could be feasible (Figure S3). The *Li*NDH2 homology model is shown schematically in Figure 3. It was further evaluated with the Ramachandran plot (Figure S4), structurally aligned and compared to the three-dimensional template structures (root-mean-square deviation (RMSD) calculations are shown in Table 1).

The structure of *Li*NDH2 contains two Rossmann domains characteristic of two-dinucleotide binding domains flavoprotein superfamily [2,7,11]. The structure differs in C-terminal domain (CTD) which is longer for *Li*NDH2 in comparison to templates, thus insufficiently modelled. The CTD is responsible for membrane interaction. The number of residues which are located in the favored region of the Ramachandran plot is 89% which includes 469 of total 527 *Li*NDH2 amino acid residues. This result was expected since the long CTD of *Li*NDH2 could not be modeled efficiently due to lack of the corresponding homology template sequence. However, none of these missing CTD residues are close to the target active site, and as such these amino acids are not relevant to our inhibitor design efforts. The number of *Li*NDH2 residues in the allowed region is 5.9%, namely 31 residue side chains are found in unusual places but have acceptable rotamers in case of loop and turn formations. Finally, the residues in the outlier region are 4.8%, meaning 25 amino acid residues are not correctly modeled. Since our interest for inhibitor design focused on the well-modeled binding site UQ_I_ of *Li*NDH2, homology modeling results were considered appropriate for structure-based design.

### 2.2. Ligand-Based Pharmacophore Modeling and Virtual Screening

**Figure 4:**
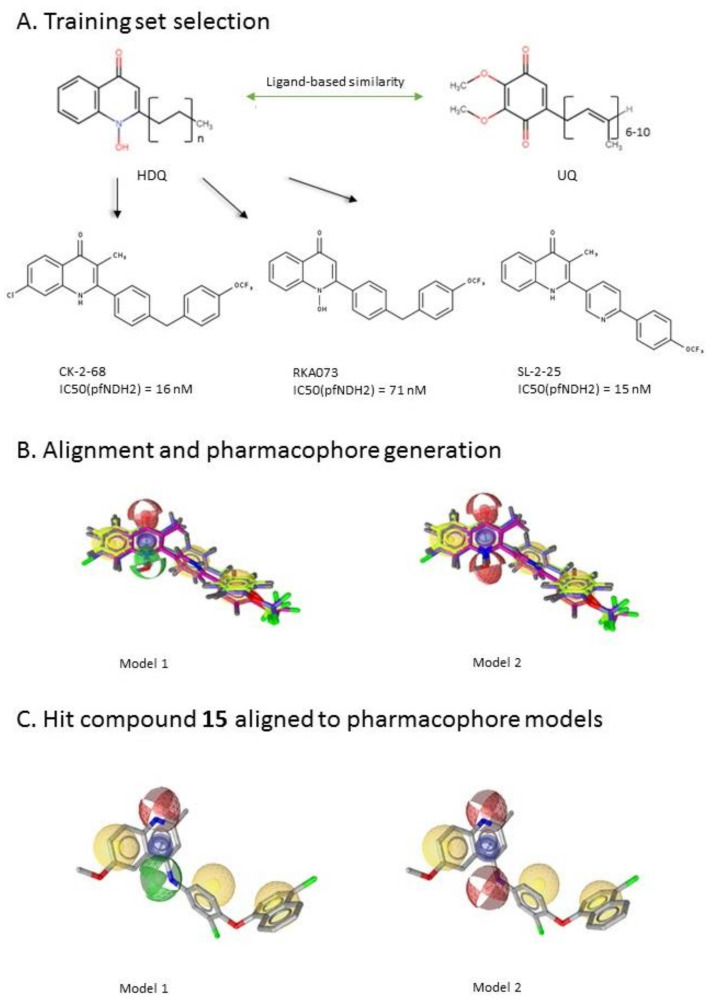
(**A**) Ligand-based similarity between UQ and hydroxy-2-dodecyl-4-(1H) quinolone (HDQ). Pharmacophore training set based on the selected HDQ derivates with IC_50_ activity against *P. falciparum*; (**B**) Derived ligand-based pharmacophore models 1 and 2 with aligned HDQ derivatives; (**C**) Example of the virtual screening hit compound **15** aligned to the pharmacophore models 1 and 2. Hydrophobic features are shown as yellow spheres, H-donor as green spheres, H-acceptors as red spheres, and aromatic feature is colored in blue.

After the generation of the *Li*NDH2 homology model, we first performed a ligand-based screening. The ligand-based pharmacophore approach is one of the standard ways to proceed with such large virtual screening and has been proved to be very efficient [21]. In the absence of the reported *Li*NDH2 inhibitors we looked for inhibitors of NDH-2s from other species. We found studies of hydroxy-2-dodecyl-4-(1H) quinolone (HDQ) derivates showing nanomolar inhibition of NDH-2 from the malaria-causing microorganism *P. falciparum* Figure 4A, [22]. These derivatives presented high chemical similarity to the ubiquinone structural scaffold that interacts with *Li*NDH2 and so we decided to test them.

Three molecules from the HDQ series were taken as starting points for further ligand-based pharmacophore design. We comprised them as a training set and generated 10 ligand-based pharmacophore models using LigandScout software [23]. We chose to proceed with the two best-ranked models to increase the coverage of the chemical space that the pharmacophore-based screening could identify. To increase the chemical space diversity, models were reduced and optimized. Reduction of the pharmacophore features was performed with the utilization of decoy compounds, molecules that were assumed to be inactive (see Methods section for further details). This has been shown to be good standard practice in the screening literature [21]. The optimized ligand-based pharmacophore model 1 has three hydrophobic elements, one H-donor, one H-acceptor and one aromatic ring pharmacophore feature, while the selected model 2 possess three hydrophobic features and two H-acceptors along with one aromatic ring pharmacophore element, as shown in Figure 4B. The two final models differ predominantly in 1 pharmacophore feature, namely substituting the H-donor (model 1) for a H-acceptor (model 2) approximately on the same geometrical position.

The in house prepared library of 500,000 commercially available compounds originating from different vendors (e.g., VITAS-M, ChemDiv, ChemBridge, etc.) was screened using both models consecutively, to discard all the compounds which were not able to align to both model’s features. Virtual screening yielded 4423 hit molecules highly diversified in their chemical structures. In Figure 4C, an example of a hit compound **15**, a 6-methoxy-quinaldine derivate, aligned to both pharmacophore models, is shown.

### 2.3. Molecular Docking of the Pharmacophore-Based Hit Compounds

We evaluated the pharmacophore-based screening hits against the *Li*NDH2 homology model by conducting a docking campaign using GOLD docking software [24]. Docking target was *Li*NDH2 UQ_I_ binding site. First, docking was performed with narrow settings of the GOLD genetic search algorithm followed by custom genetic algorithm (GA) flexibility settings in order to explore conformational space within UQ_I_. We reduced the number of hits for further analyses by considering only those hits with GoldScore scoring function values between 60–90 as the optimal docking scores for meaningful docking results [24].

**Figure 5:**
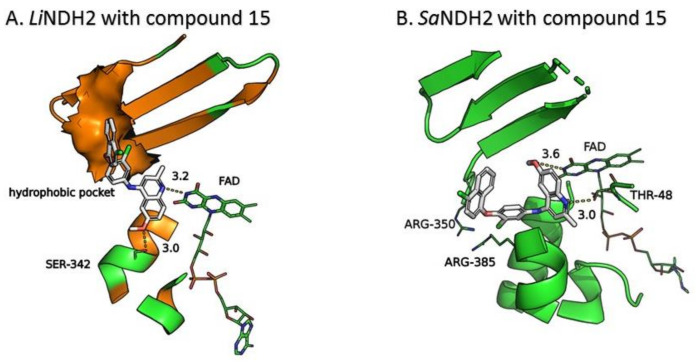
Proposed binding modes of the substituted 6-methoxy-quinaldine compound **15** at (**A**) *Li*NDH2 UQ_I_ binding site; and (**B**) *Sa*NDH2 UQ binding site.

This criterion was satisfied by 54 compounds; for example, for compound **15** the proposed binding mode in the *Li*NDH2 UQ_I_ binding site is depicted in Figure 5A. The binding mode, shows a formation of H-bonding with the isoalloxazine ring of FAD and Ser324, which is also observed in the case of UQ. Furthermore, the chloronapthalen-1-yl-oxyphenyl moiety of compound **15** fills the hydrophobic pocket (Figure 5A, colored in orange) comprised by a α-helix (Ala397–Leu407) and β-sheet (Gly371–Leu385). Predicted affinity with GoldScore value of 79.25 is also fully comparable with that of UQ docking, which scores between 80–84 depending on the conformation of the UQ hydrophobic chain.

Comparison of the crystallographic structure of the UQ binding pose (*Sc*NDH2), re-docked UQ into template structure (*Sc*NDH2) and docked UQ into the model (*Li*NDH2), allowed us to explore the conditions for the docked compounds. First, amide nitrogen of the isoalloxazine ring of FAD acts as H-donor and should interact with the H-acceptor from the active hit compounds. Second, the hydroxyl group from the amino acid residue Ser324 should interact with the hit compounds through formation of a hydrogen bond or OH–π interaction. In some cases Tyr25, which is positioned close to Ser324, contributes to the stabilization of the compounds in the binding site mainly through hydrogen bonding. The hypothesis for the importance of these interactions is further supported by the docking of the UQ in the mutant models of *Li*NDH2 without Ser324 and Tyr25, (Figure S5). Finally, hit compounds should fill the hydrophobic pocket formed between α-helix (Ala397–Leu407) and β-sheet (Gly371–Leu385). These guidelines were used to predict potential interactions and to reduce the number of selected hit compounds.

We further assumed that docking poses in *Li*NDH2 UQ_I_ binding site should align with proposed pharmacophore model features in regards to orientation and position in the binding site. It was gratifying to observe that the majority of docked conformations of the selected compounds strongly resembled the alignment with pharmacophore-based models, pointing to specific ligand-protein interactions (see Figure S6, example with compound **15**). Also, we considered diversity among scaffolds for further testing and excluded potential pan-assay interference compounds (PAINS) [25]. In the final compound selection, we also considered pharmacophore scoring function values of compounds aligned to our pharmacophore (PharmFit), used GoldScore scoring function values for docking modes and a geometrical consensus between the pharmacophore fitting of hits to the pharmacophore models and docking binding modes in the *Li*NDH2 UQ_I_ binding site (see Table S2 for score values). Finally, we selected 23, chemically very diverse hit compounds for experimental testing (Table 2). Sources of all commercially available compounds are provided in supplementary information Table S3.

### 2.4. Results of the In Vitro Inhibition Assays and Measurements of the Leishmanicidal Effect

Selected compounds were first assayed for their in vitro inhibition activity in the structurally similar recombinant NDH-2 from *S. aureus* (*Sa*NDH2), since isolated *Li*NDH2 was not available. Most of the selected compounds covering a large conformational space showed only negligible inhibition activity (Table 2). This reflects the complex nature of the ligand–protein molecular recognition process that poses challenges to full explanation due to lack of data, known limitations in screening models, and diversity among enzymes. 

**Figure 6:**
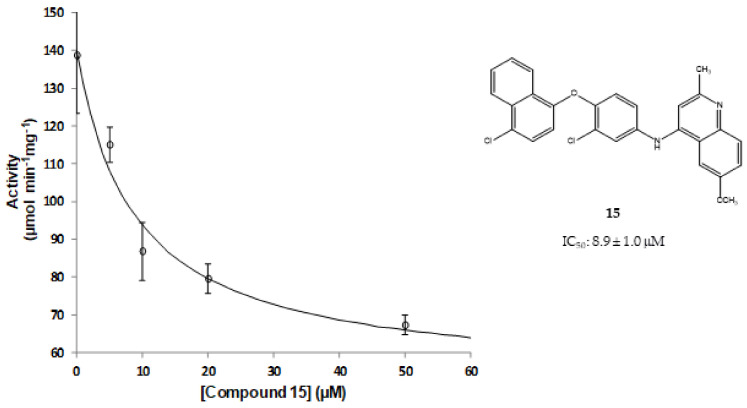
Steady-state analyses of the activity of NDH-2 from *S. aureus* in the presence of compound **15**.

Nevertheless, compound **15**, a substituted 6-methoxy-quinaldine, did show promising inhibition results for *Sa*NDH2 in the initial screening with the RA value of 49% measured at 20 µM. Binding modes of the hit compound **15** to *Sa*NDH2 and *Li*NDH2 both show H-bonds with FAD and, additionally, interaction with Thr48 side chain is recorded in case of *Sa*NDH2 binding (Figure 5B). Alternative conformations of the 6-methoxy-quinaldine scaffold pose are observed among the docking results. This indicates that the mechanism of binding for compound **15** as a general NDH-2 inhibitor is debatable, although correct according to the binding hypothesis. Encouraged by this positive result, we preceded with *K_i_^app^* determination using steady-state analyses which resulted in the 8.9 ± 1.0 µM value. This classifies compound **15** as a promising hit due to its low micromolar range inhibition activity (Figure 6). It should be noted that for compound **15** anti-malarial activity was previously reported [26,27,28].

**Figure 7:**
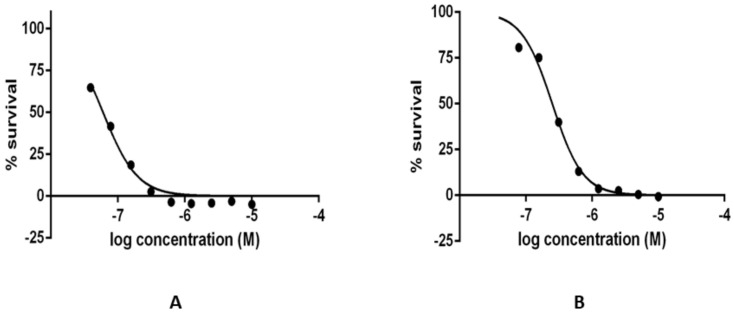
Concetration-dependant leishmanicidal effect in wild type axenic (**A**) proamastigotes and (**B**) amastigotes in *L. infantum* cultures for compound **15**.

We were further interested to know how much the compounds could influence the growth of the *L. infantum* parasite. Two randomly selected inactive compounds from the in vitro enzyme inhibition assay, compounds **11**, **20** along with the active compound **15**, were selected to assay their leishmanicidal activity over wild-type *L. infantum* axenic amastigotes and promastigotes. The selection of inactive compounds was done also to probe whether the observed inactivity at the in vitro level would be noticed at the level of the leishmanicidal determination. Graphs of the compound’s typically observed leishmanicidal effect in the wild-type axenic proamastigotes and amastigote cultures for the hit compound **15** are shown in Figure 7.

Only compound **15** that showed strong in vitro inhibition of the NDH-2 enzyme (*Sa*NDH2) also possessed leishmanicidal activity with the potent IC_50_ values in the interval between 0.03–0.05 µM against promastigotes and in the interval between 0.2–0.3 µM against amastigotes of *L. infantum* (Table 3 and Figure 7). Interestingly, compound **15** in previous investigations of leishmanicidal activity against transgenic *Leishmania mexicana* promastigotes that were glucose-transport deficient also showed some potential [29]. Thus, compound **15** displays itself as a promising starting point for further development of potent agents against *Leismania infantum* parasites. One important additional aspect that will have to be evaluated in further development of this hit compound is also to determine its influence on the mammalian cells [30].

## 3. Materials and Methods

### 3.1. Construction of the LiNDH2 Homology Model

Protein homology/analogy recognition engine [14], Phyre 2 (v2.0) provided a possibility for the *Li*NDH2 template proteins’ identification and homology modelling in order to carry out rational structure-based design in search for novel inhibitors in the absence of the *Li*NDH2 crystal structure. The sequence of *Li*NDH2 found under entry A4IDV2 from UniProt web server [31], was used as a query to Phyre 2 server with normal modelling mode. The best template structures identified were *Sc*NADH:ubiquinone oxidoreductase (Chain B, PDB: 4g6g or 4g73) and *Sa*NADH:quinone oxidoreductase (Chain C, PDB: 4xdb) with 33% and 27% identity, respectively, and 100% modelling confidence [9,15,16]. The template sequences, *Sc*NDH2 (P32340), *Pf*NDH2 (Q8I302) and *Sa*NDH2 (Q2FZV7) were retrieved for alignment purposes. The sequence alignment was generated using Clustal X software and comparison between sequences of corresponding structures was done via Sequence Manipulation Suite which provided sequence similarity and homology similarity between templates and *LiNDH2* query sequence [20,32]. This was done afterwards to further explore amino acid sequence similarities due to automatic template utilization using the Phyre 2 server. Furthermore, the homology model was evaluated using the RAMPAGE tool through generation of a Ramachandran plot [33]. For additional information regarding the homology model using Ramachandran plot, see the supplementary material (Figure S4).

The structure of *Sc*NDH2 (PDB ID 4g73) was downloaded and prepared for the analysis of the crystallographic UQ binding site. Pymol software was used to remove undesired components (“A” chain, crystal waters, other molecular residues) except chain “B”, containing UQ, FAD, NADH and Mg^2+^ metal ions. Crystallographic structural data was used for the extraction of FAD, NADH and UQ conformations and poses in order to set up docking parameters with evaluation of re-docking results using RMSD.

### 3.2. Alignment of Protein 3D Structures

We aligned the *Li*NDH2 homology model and two templates using pymol software [34]. Both templates: (1) *Sc*NDH2 (PDB ID 4g73) and (2) *Sa*NDH2 (PDB ID 4xdb) were downloaded into pymol and cleaned through removal of non-protein atoms and chains until mono units were obtained. Afterwards, we optimized the CTD of the *Li*NDH2 homology model for alignment purposes by truncating it at residue 436 (Table S1). RMSD values were calculated along the alignment in two steps: (1) RMS for all atoms and (2) RMSD for the C-backbone only. Not all of the residues were aligned and certain gaps were observed; thus the final alignment was calculated in percentages of total 436 residues of the model (with 436 being considered as 100%). The results are summarized in Table 1. Three-dimensional structural alignment was used to generate molecular data (system size, conformations and position of cofactor and ligands) needed for structure-based design.

### 3.3. Ligand-Based Pharmacophore Modeling and Virtual Screening

Ligand-based pharmacophore modeling and virtual screening were performed using LigandScout [23]. The ligand-based pharmacophore models were built using available data of the selected HDQ derivatives with nanomolar inhibition activity on NDH-2 from the malaria-causing microorganism *P. falciparum*. Using ChemDraw, we draw the 2D structures of three selected HDQ derivatives [35]. We optimized and minimized their three-dimensional structures in LigandScout’s implemented MMFF94 force field, and imported them into the ligand-based module within LigandScout for ligand-based pharmacophore generation.

Parameters for the LB conformer generator in OMEGA software were set to obtain 500 unique conformations for each of the active compounds. The RMS threshold for duplicate conformers was set to 0.4 Å, the maximum number of all generated conformers per molecule to 30,000, and the maximum number of intermediate conformers per molecule to 4000 [36]. The conformations were then dynamically aligned yielding 10 final ligand-based merged pharmacophore models. We used the LigandScout inbuilt scoring function that combined pharmacophore fit and atom shape overlap in order to assess the models produced. The LB pharmacophore models with the highest score of 0.911 and 0.910 were selected for further use, Figure 4B,C.

For the chemical space diversity search and recognition patterns, our chosen pharmacophore models were optimized in a number of available merged pharmacophore features. The discriminatory performance of the derived pharmacophore models was validated by a screening experiment against 150 decoys (50 per selected active compound) using a Database of Useful Decoys: Enhanced (DUD-E server) [37]. Decoys were generated by introducing SMILES of active compounds as template molecules into DUD-E server. Decoys have similar physical properties but different chemical structures, which is defined by the maximum Tanimoto value threshold between active compound and decoy molecule MACCS fingerprints [37]. The decoys were employed in small benchmarking screening procedures over a total of 10,000 compounds from home libraries with potential activity. Pharmacophore models were run in a subsequent manner. Screening with model 1 coupled with subsequent screening with model 2 reported 24/150 decoys in the first cycle run. When the reduction and modification of pharmacophore features in model 1 and 2 took place, identical screening procedure yielded 3/150 decoys among a final training set of 1186 potential hits. The purpose of this training method was to optimize the number of pharmacophore features of the models in the lack of known *Li*NDH2 active compounds. The reduced ligand-based pharmacophore models were used in the virtual screening process. The number of compounds in the used virtual library was approximately 550,000. Screening libraries contained commercially available compounds converted into the multifunctional format, 25 conformations for each compound in the library. The screening algorithm took approximately 17 h to complete.

### 3.4. Molecular Docking Calculations

For molecular docking purposes, we used a standard GOLD docking program coupled with the GoldScore fitness function [24]. Docking calculations were performed in the presence of FAD and NADH that we modeled in the *Li*NDH2. Cofactor and substrate structures were obtained from PDB 4g73 prior to the docking calculation after 3D structural alignment. We first optimized docking settings by docking the UQ molecule into template structures *Sc*NDH2 and *Sa*NDH2. Then, we docked the UQ *Li*NDH2 homology model in order to assess the docking performance and compare the UQ posing results with the crystallographic data. Protein structures were prepared in mol file format as suggested in the GOLD manual [24]. The following settings were used: defined binding site as all atoms within 6 Å of the crystallographic UQ, that is within the UQ_I_ binding site, and we defined a constraint of 3.5 Å between residues Leu444 and Leu447 of the pdb ID 4g73 structure. This constraint gave an additional score for the flexible hydrophobic moiety of UQ, which is observable in the crystallographic ligand pose in the template (4g73). We used the same settings for the docking of UQ in the *Sa*NDH2 and *Li*NDH2 models with a constraint within the hydrophobic cavity (for the homology model, the constraint sphere encompasses the residues between Arg368–Met373 and Val383–Leu385). The following docking experiments were performed: (1) redocking of the UQ molecule into the template structure (PDB ID 4g73); (2) docking of UQ into the *Sa*NDH2 template structure (PDB ID 4xdb); (3) docking of UQ, active compounds (HDQ derivatives used for ligand-based pharmacophore design); and (4) docking of hit compounds obtained from pharmacophore-based virtual screening into the *Li*NDH2 model UQ_I_ binding site. Docking procedures (1), (2) and (3) were carried out using the GA with 120% search efficiency. We calculated the RMSD between the crystal UQ (4g73), re-docked UQ (4g73) and UQ docked into the *Li*NDH2 model. The calculated average RMSD of the UQ’s aromatic core between crystallographic and multiple docked UQ poses was 1.740 Å and an average GoldScore of 82.8 was obtained. (Table S4).

Docking evaluation (d) of 4423 pharmacophore-based screening hit compounds was done subsequently, using the GA of GOLD with virtual screening settings, a cut-off set to 50 and a maximum number of conformations per molecule equal to 3. We obtained 13,041 docking conformations out of these pharmacophore-based hits which were visualized and evaluated using LigandScout. Subsequently, the best 428 hits were re-docked using the GA algorithm with 120% search efficiency (flexible settings). Poses having docking GoldScore between 60–90 (presumed as optimal scoring function values) were kept and were visually examined and compared to the UQ docking reference. A total of 54 hits (318 conformations) were submitted for final filtering and selection (see Results and Discussion section) for activity evaluation. These 54 hits were also docked to *Sa*NDH2 using AutoDock Vina with exhaustiveness = 100, maximum number of docking modes = 10, and maximum energy difference = 3 prior to experimental testing on *Sa*NDH2 [38].

### 3.5. Steady-State Kinetics Experiments to Determine Inhibition Activity of NDH-2 from S. aureus

NADH was purchased from Sigma Aldrich. 2,3-Dimethyl1,4-naphthoquinone (DMN) was synthesized from menadione (Sigma Aldrich, St. Louis, MO, USA) [39]. In order to test the effect of the studied compounds **1**–**23**, activity assays were performed on a Shimadzu UV-1800 spectrophotometer monitoring the change in absorbance of the electron donor, NADH, at 340 nm [15]. The activity assays were made inside an anaerobic chamber at 35 °C, using DMN as electron acceptor. The NADH extinction coefficient of 6.22 mM^−1^ cm^−1^ was used to calculate NDH-2 specific activity (μmol^−1^ min^−1^ mg protein). The reaction mixture (1000 μL) contained 20 nM NDH-2 in 100 mM potassium phosphate pH 7.0, 250 µM NaCl, 100 μM NADH, 150 µM DMN and the tested compounds were added to the reaction mixture at the final concentration of 20 µM. Residual activity (RA) values were calculated by using the determined NDH-2 activity at the investigated inhibitor concentration divided by the NDH-2 activity determined in the absence of the inhibitor. For compound **15**, the Ki determination was performed at the following concentrations, 50 µM, 20 µM, 10 µM and 5 µM, using the same procedure described. The obtained data points were fitted using equation:

(1) $$v_{0}^{i}=v_{0}^{max}\frac{\left(1+\beta \frac{\left[l\right]}{K_{i}^{app}}\right)}{\left(1+\frac{\left[l\right]}{K_{i}^{app}}\right)}$$

to calculate the apparent *K_i_^app^* of 8.9 ± 1.0 µM.

### 3.6. Determination of Selected Compound Leishmanicidal Effect in Axenic Amastigotes of L. infantum

*L. infantum* promastigotes (MHOM MA67ITMAP263) were cultured at 25 °C in RPMI 1640 Glutamax supplemented with 10% (*v*/*v*) inactivated fetal bovine serum (iFBS), 50 U mL^−1^ penicillin, 50 mg mL^−1^ streptomycin (from Gibco) and 25 mM HEPES sodium salt pH 7.4 (Sigma). Axenic amastigotes of *L. infantum* were grown at 37 °C and 5% CO_2_, in MAA medium supplemented with 20% (*v*/*v*) iFBS, 2 mM Glutamax (GibcoBRL), and 0.023 µM hemin (Sigma), as described previously [40]. Parasites were seeded at 1.5 × 10^6^ cells mL^−1^ in 96-well plates in complete MAA20 medium for amastigotes and RPMI for promastigotes with varying concentrations of the different compounds. Twenty-four hours later, parasite viability was measured by the resazurin assay and calculated as the percentage in relation to control cultures incubated with the vehicle alone, as described [41]. Data were analyzed and half-maximal inhibitory concentration (IC_50_) values determined.

## 4. Conclusions

There is an urgent need for new antileishmanial drugs with alternative unexploited mechanisms of action for tackling widespread leishmaniases. Alternative NADH dehydrogenase from *L. infantum* is an important new target, its inhibition expecting to cause respiration difficulties leading to the possible elimination of the targeted parasite [4]. Based on the available structural data from NDH-2s from *S. cerevisiae* and *S. aureus*, we developed and validated a three-dimensional homology model of the *L. infantum* NDH-2. Then, ligand-based pharmacophore models and virtual screening, using reported 4-quinolone inhibitors of NDH-2 from *P. falciparum*, were coupled with subsequent molecular-docking calculations, which resulted in 54 hits with an inhibitory potential. Twenty-three compounds displaying a consensus of docking scores were selected, and pharmacophore fit scores and visual binding modes were assayed for their in vitro inhibitory activity towards recombinant NDH-2 from *S. aureus* and their leishmanicidal activity for *L. infantum* axenic amastigotes and proamastigotes. Our experimental results identified 6-methoxy-quinaldine as an inhibitor of NDH-2 from *S. aureus* (*K_i_^app^* = 8.9 ± 1.0 µM). This compound also exhibits a promising leishmanicidal effect in the nanomolar range in wild-type axenic amastigotes (IC_50_ interval between 200–300 nM) and promastigotes (IC_50_ interval between 30–50 nM) rendering it of potential interest for further development and optimization. Our combined results comprise a valuable step towards the discovery of resistance-free NDH-2 inhibitors and the resulting compound **15** could serve as a starting point in developing a potent drug against *Leismania infantum* parasites.

## Figures and Tables

**Table 1:** Results of the 3D structural alignment between the *Li*NDH2 homology model and the templates used—*Sc*NDH2 (4g73) and *Sa*NDH2 (4xdb), RMSD is expressed as all atoms and Cα-chain (backbone).

Root-Mean-Square Deviation (RMSD) for	Model to 4g73 (No. of Residues and % Aligned)	Model to 4xdb (No. of Residues and % Aligned)
All atoms	0.227 Å (321 residues, 74% aligned)	2.822 Å (370 residues, 85% aligned)
Cα-chain	0.923 Å (396 residues, 91% aligned)	1.331 Å (252 residues, 58% aligned)

**Table 2:** Results of the inhibition assays of the selected hit compounds **1**–**23** derived from the virtual screening campaign against *Li*NDH2, expressed as relative activity (RA) of the NDH-2 from *S. aureus*. RAs in % at 20 µM compound concentration were calculated considering the activity in the absence of any inhibitor as 100%.

Compd.	Chemical Structure	RA	Compd.	Chemical Structure	RA	Compd.	Chemical Structure	RA
**1**	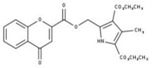	93%	**9**	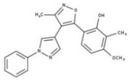	80%	**17**	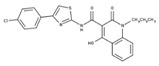	91%
**2**	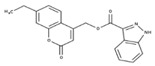	75%	**10**	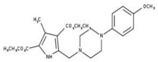	92%	**18**	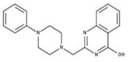	86%
**3**	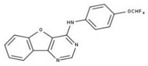	71%	**11**	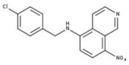	83%	**19**	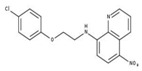	92%
**4**	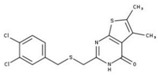	74%	**12**	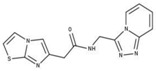	95%	**20**	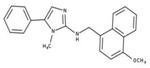	84%
**5**	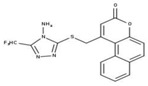	86%	**13**	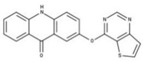	86%	**21**	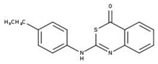	94%
**6**	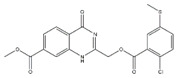	89%	**14**	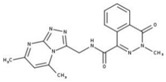	93%	**22**	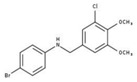	86%
**7**	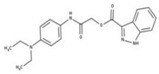	86%	**15**	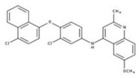	49%	**23**	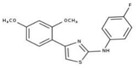	88%
**8**	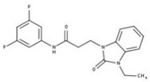	99%	**16**	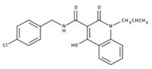	87%			

**Table 3:** Results of the leishmanicidal effect measurement of selected hit compounds in the wild-type axenic amastigotes and promastigotes of *L. infantum.*

Compound	Wild Type IC_50_ (µM)	
	Promastigotes	Amastigotes
**11**	5–10	>20
**15**	0.03–0.05	0.2–0.3
**20**	5–10	>20

Provided intervals are results of three independent measurements.

## Supplementary Materials

The following are available online.

## Abbreviations

The following abbreviations are used in this manuscript:

CTD	C-terminal domain
FAD	Flavin adenine dinucleotide
GA	Genetic algorithm
HDQ	hydroxy-2-dodecyl-4-(1H) quinolone
LiNDH2	*Leishmania infantum* NDH-2
NADH	Nicotinamide adenine dinucleotide
NDH-2	Alternative (or type 2) nicotinamide adenine dinucleotide dehydrogenase
PfNDH2	*Plasmodium falciparum* NDH-2
PAINS	Pan assay interference compounds
PDB	Protein data bank
RMSD	Root-mean-square deviation
ScNDH2	*Saccharomyces cerevisiae* NDH-2
SaNDH2	*Staphylococcus aureus* NDH-2
UQ	Ubiquinone
UQ_I_	1st Ubiquinone binding site
UQ_II_	2nd Ubiquinone binding site
