# Supplementary material for: In Silico Discovery of a Substituted 6-Methoxy-quinaldine with Leishmanicidal Activity in Leishmania infantum
Source: Molecules. 2018 Mar 27;23(4):772. doi: 10.3390/molecules23040772 (PMC6017605; doi:10.3390/molecules23040772)
Supplement: Supplementary file 1 [file molecules-23-00772-s001.pdf]

molecules

Supplementary Material:

In Silico Discovery of a Substituted 6-Methoxy-quinalidine with Leishmanicidal Activity in *Leishmania infantum*

Strahinja Stevanović[1]*, Andrej Perdih[2]*, Milan Senćanski[1], Sanja Glišić[1], Margarida Duarte[3], Anna Tomás[3], Filipa V. Sena[4], Filipe M. Sousa[4], Manuela M. Pereira[4, 5] and Tom Solmajer[2]

[1] *Center of Multidisciplinary Research, Institute of Nuclear Sciences "Vinča", University of Belgrade, 11001 Belgrade, Serbia*
[2] *National Institute of Chemistry, Hajdrihova 19, 1001 Ljubljana, Slovenia*
[3] *i3S-Instituto de Investigação e Inovação em Saúde, Universidade do Porto and IBMC-Institute for Molecular and Cell Biology, 4099-002 Porto, Portugal*
[4] *ICBAS, Instituto de Ciências Biomédicas Abel Salazar, Universidade do Porto, 4099-002 Porto, Portugal*
[5] *Instituto de Tecnologia Química e Biológica António Xavier, Universidade Nova de Lisboa, 1099-085 Oeiras, Portugall*
[6] *University of Lisbon, Faculty of Sciences, BioISI-Biosystems & Integrative Sciences Institute, Campo Grande, C8,1749-016 Lisboa, Portugal*
* Correspondence: sstevanovic@vin.bg.ac.rs;
* Correspondence: andrej.perdih@ki.si; Tel.: +386-1-4760-376

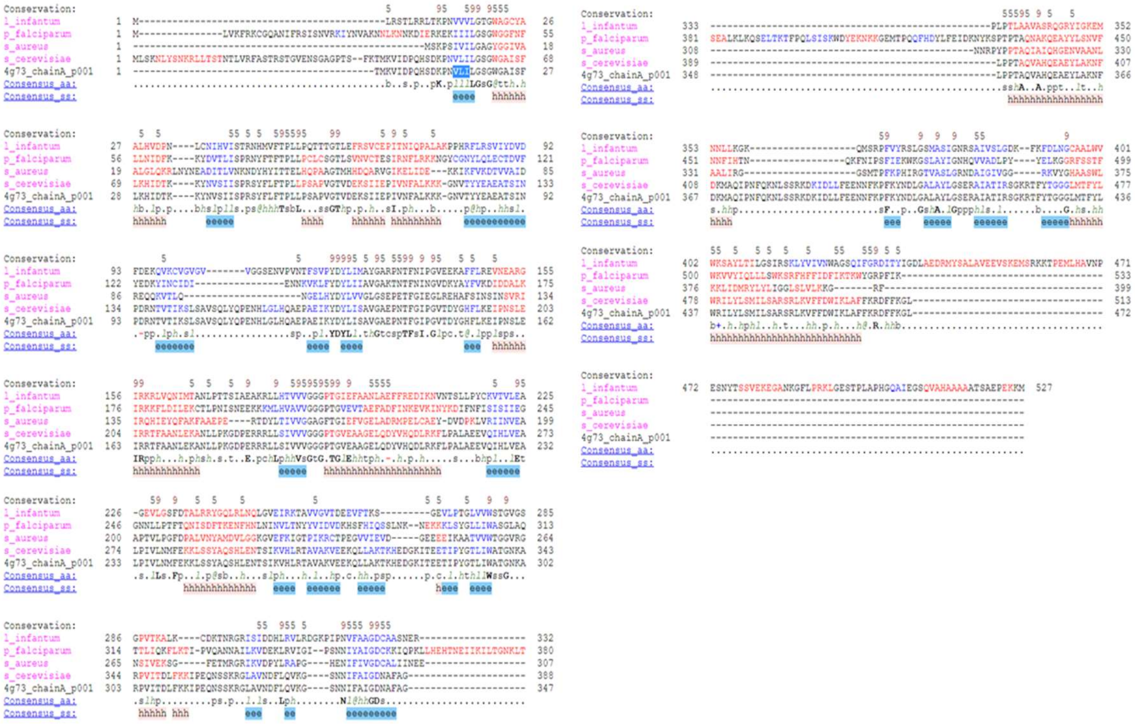

Figure S1. Secondary Structure Alignment generated from LiNDH2, ScNDH2, SaNDH2 and PfNDH2 sequence alignment using PROMALS3D: a tool for multiple sequence and structure alignment. For the reference template —PDB ID 4g73 is used (available crystal structure of ScNDH2). In consensus secondary structure line: With letter "h" chain—alfa-helix is marked, and with "e" chain, beta-sheet.

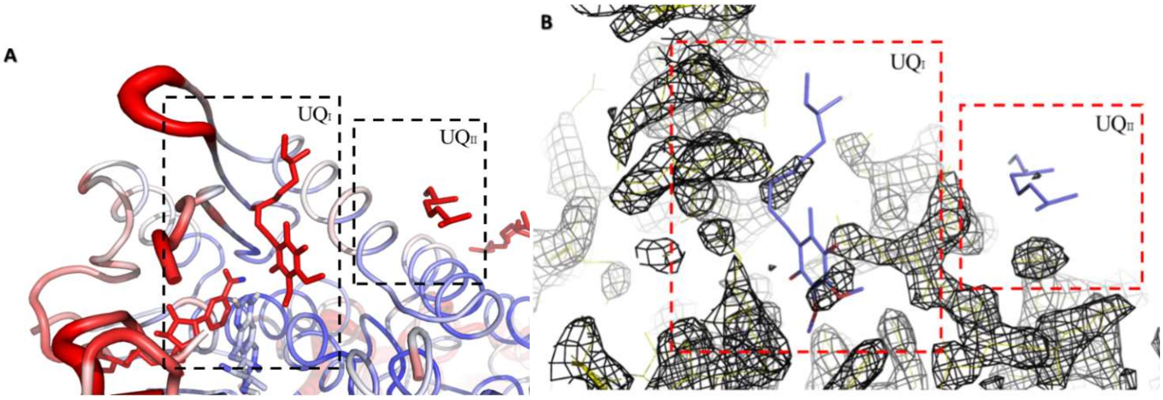

Figure S2. **(A)** B-factor representation of S. *cerevisiae* NDH-2 (PDB ID: 4G73) focused on binding site—UQI which contains UQ, FAD and NADH as a form of ternary complex. Color code: white(high)-to-red(low) quantification of atom position certainty, traced using pymol software. **(B)** Isomesh displayed using pymol's CCP4—format electron density mapping. Lower electron density population is shown in the region of second—UQII binding site, thus making it difficult to deduce conformational occupancy of UQ and amino acid residues from X-ray structure.

Table S1. Homology models generated using Phyre 2 server. First two homologues structural models are shown;

Template	Confidence	% i.d.	Template Information
4G6G_B<br>4G73_B<br>**(same seq.)**	100.0	33	**PDB header:** oxidoreductase<br>**Chain:** B: **PDB Molecule:** rotenone-insensitive nadh-ubiquinone oxidoreductase,<br>**PDBTitle:** crystal structure of NDH-2 with TRT<br>**Resolution:** 2.5 Å
4XDB_C	100	27	**PDB header:** oxidoreductase, membrane protein, flavoprotein<br>**Chain:** C: **PDB Molecule:** nadh dehydrogenase-like protein saouhsc_00878;<br>**PDBTitle:** nadh:quinone oxidoreductase (ndh-ii) from staphylococcus aureus -2 holo protein structure.<br>**Resolution:** 3.32 Å
Note: For both templates alignment coverage is around 80 %			

Table S2. Obtained pharmacophore and docking scores for twenty-three selected hit compounds.

No.	Scaffold	Pose/conformation	PharmFit	GOLD.fitness
1	PYRROLE	1	48.2	78.9353
		2	48.2	78.8489
		3	48.2	73.5001
2	CHROMEN	1	47.4	71.5822
		2	47.4	71.1047
		3	47.4	67.7467
3	benzofuro[3,2-d]pyrimidin	1	46.62	71.1031
		2	46.62	67.5164
		3	46.62	67.4288
4	THYENO[3,2-d]PYRIMIDIN	1	49.46	67.0032
		2	49.46	66.7081
		3	49.46	64.9063
5	CHROMEN	1	47.02	70.0423
		2	47.02	67.3608
		3	47.02	66.6311

6	quinazoline	1	48.01	71.0211
		2	48.01	70.8991
		3	48.01	69.7889
7	INDAZOLE	1	47.28	74.1601
		2	47.28	72.2617
		3	47.28	60
8	BENZYMIDAZOLE	1	46.25	73.6621
		2	46.25	71.6988
		3	46.25	65.6762
9	O,M-METOXY	1	49.57	66.1606
		2	49.57	66.0541
		3	49.57	66.189
10	PYRROLE	1	47.41	83.8593
		2	47.41	82.4897
		3	47.41	80.6411
11	nitroisoquinoline	1	46.43	69.5886
		2	46.43	68.1004
		3	46.43	66.7446
12	TRIAZOLO[4,3-a]PYRIDIN	1	46.83	72.574
		2	46.83	70.5994
		3	46.83	60
13	THYENO[3,2-d]PYRIMIDIN	1	46.33	73.0864
		2	46.33	72.0532
		3	46.33	69.4898
14	dihydrophthalazine	1	50.63	72.2082
		2	50.63	71.9494
		3	50.63	71.0051
15	quinoline	1	47.45	79.2522
		2	47.45	77.9944
		3	47.45	77.1914
16	quinazoline	1	47.72	77.3913
		2	47.72	75.1822
		3	47.72	71.6327
17	quinoline	1	49.78	74.6217
		2	49.78	74.0581

		3	49.78	73.5339
18	QUINAZOLINE	1	49.8	70.3329
		2	49.8	69.9571
		3	49.8	68.429
19	nitroquinoline	1	49.45	74.248
		2	49.45	73.1649
		3	49.45	69.6986
20	naphthalene	1	46.1	73.5739
		2	46.1	72.3605
		3	46.1	60
21	benzothiazine	1	46.04	64.2407
		2	46.04	63.8897
		3	46.04	62.9287
22	aniline	1	46.53	63.8863
		2	46.53	61.1532
		3	46.53	60
23	pyrrole	1	47.52	71.4297
		2	47.52	70.6303
		3	47.52	60
REF	ubiquinone	1	50	80-84
		2	50	80-84
		3	50	80-84

Table S3. Selected commercially available compounds derived from ligand-based virtual screening campaign against *Li*NDH2.

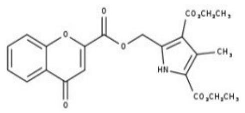 **1** (T5251861)	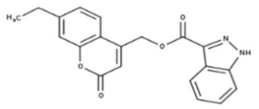 **2** (T5338693)	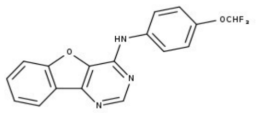 **3** (T5357635)
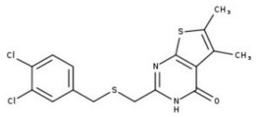 **4** (T5418152)	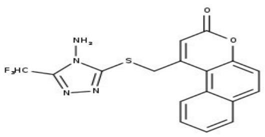 **5** (T5510138)	**6** (T5550562)

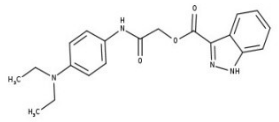 **7** (T5826550)	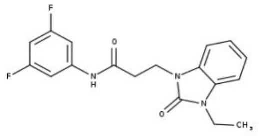 **8** (T5925359)	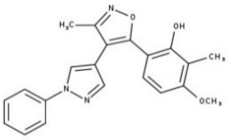 **9** (T5268007)
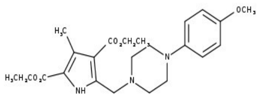 **10** (T5368230)	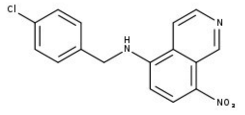 **11** (T5320777)	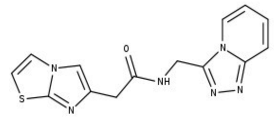 **12** (T5803358)
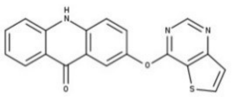 **13** (T5811637)	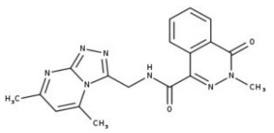 **14** (T5973959)	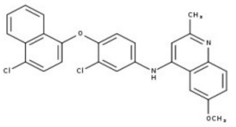 **15** (STK061205)
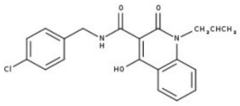 **16** (STK047376)	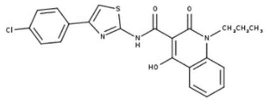 **17** (STK002301)	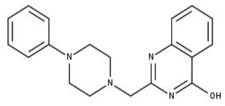 **18** (STK149674)
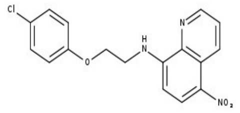 **19** (STK085541)	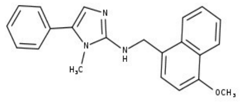 **20** (STK118160)	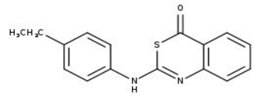 **21** (STK042244)
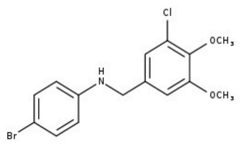 **22** (STK140911)	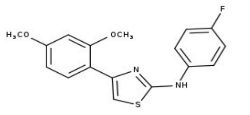 **23**(T5330742)	

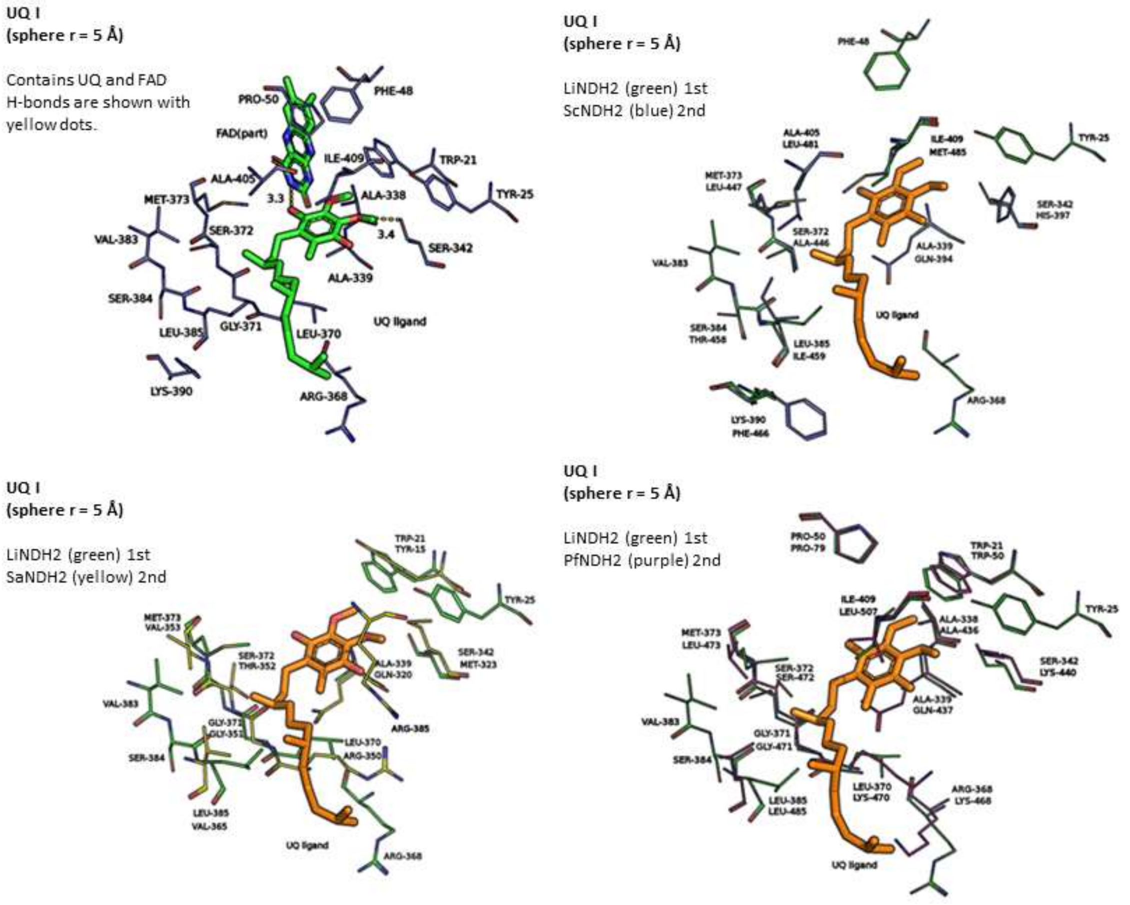

Figure S3. Binding site comparison of different NDH-2s. Upper left—binding site UQ_I in the model *Li*NDH2 showing UQ molecule and important residues. Upper right—comparison of UQ_I between *Li*NDH2 and S*c*NDH2. Lower left—comparison of UQ_I between *Li*NDH2 and S*a*NDH2. Lower right—comparison of UQ_I between *Li*NDH2 and *Pf*NDH2. For clarity reasons, only the differences in amino acid between structures are shown in comparisons.

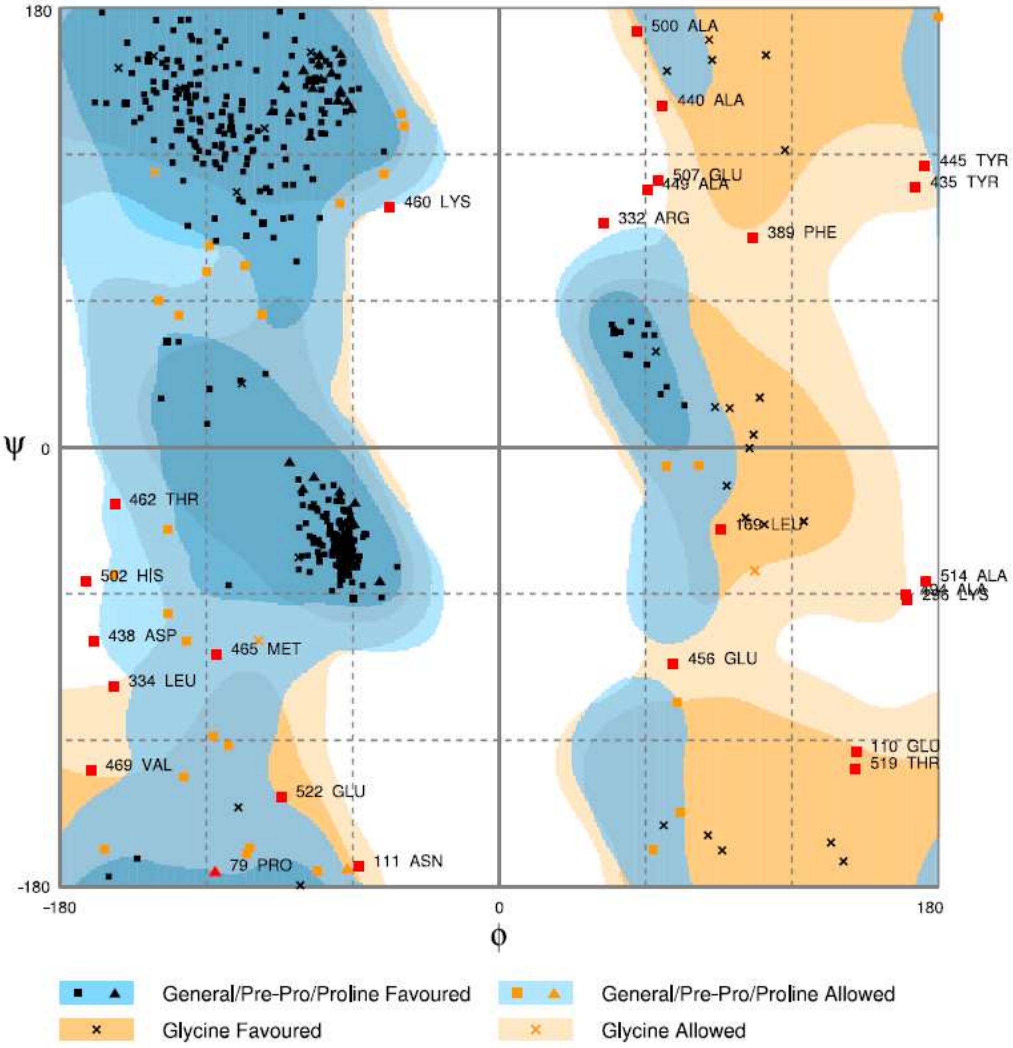

Figure S4. Ramachandran plot for *Li*NDH-2, calculated using RAMPAGE.

Table S4. Docked UQ in homology structure of *Li*NDH2 against UQ experimental posing from template structure 4G73. RMSD value refers to conformational differences in active site features, amino acid environment.

Docked UQ vs reference—UQ experimental	RMSD (Å)
ǀUQǀdock 1—reference	1.9159
ǀUQǀdock 2—reference	1.9501
ǀUQǀdock 3—reference	1.2439
ǀUQǀdock 4—reference	1.7549

Testing Hypothesis

GOLD Fitness Score

H1 (Ser342, Tyr25)	Ho
64.1708	66.303
61.1928	65.8198
66.7914	63.7807
66.8341	63.2555
65.5948	62.5137
65.4237	60.5077
65.7175	59.7846
65.6428	58.5946
...	...

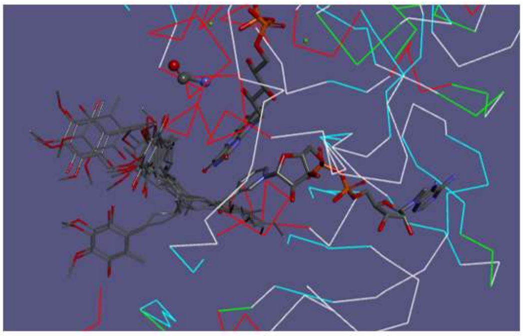

Figure S5. Results of testing of the docking modes of UQ without Ser324 and Tyr25

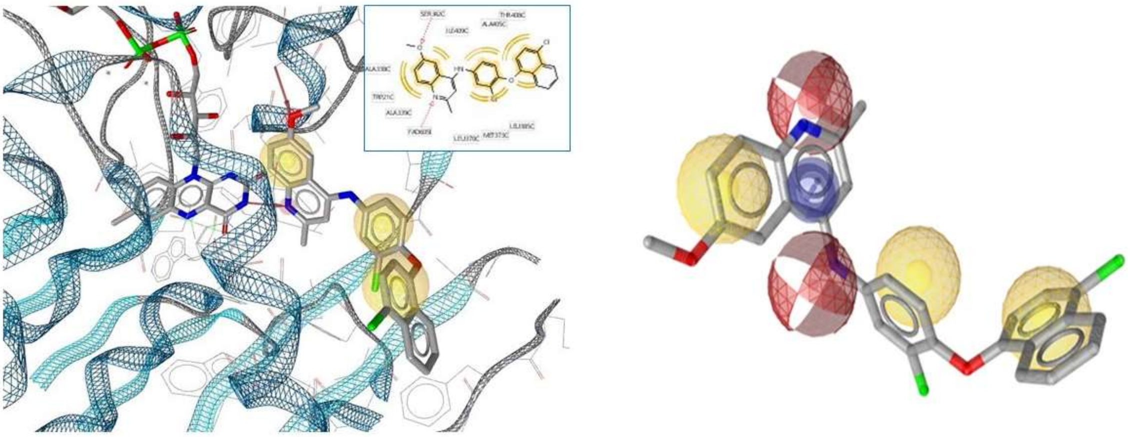

3D Structure-based pharmacophore model generated from docked compound **15** docked into LiNDH2 Active Site (UQ$_i$).

3D Ligand-based pharmacophore model 2 with screening result compound **15** aligned.

Figure S6. Proof of structure-based and ligand-based consensus in case of compound **15**. Pharmacophore model generated from active compounds predicts functional group composition as a blueprint necessary for interaction with *Li*NDH2 structure.
